# Long‐term impact of Elexacaftor/Tezacaftor/ivacaftor on pulmonary, nutritional and metabolic outcomes in homozygous F508del cystic fibrosis patients: A real‐world cohort study

**DOI:** 10.1002/bcp.70460

**Published:** 2026-01-23

**Authors:** Nicola Perrotta, Luigi Angelo Fiorito, Roberta Vescovo, Anna Virgilio, Giulia Amato, Giuseppe Cimino

**Affiliations:** ^1^ Department of Physiology and Pharmacology “V. Erspamer” Sapienza University Rome Italy; ^2^ Policlinico Umberto I Hospital Sapienza University Rome Italy; ^3^ Department of Chemistry and Technology of Drugs Sapienza University Rome Italy; ^4^ Regional Cystic Fibrosis Center, Policlinico Umberto I Hospital Sapienza University Rome Italy

**Keywords:** cystic fibrosis, Elexacaftor/Tezacaftor/ivacaftor, inflammation, liver function, metabolic health, nutritional status

## Abstract

**Aim:**

Elexacaftor/tezacaftor/ivacaftor (ETI) has markedly improved cystic fibrosis (CF) outcomes. However, its long‐term impact on nutrition, metabolism and liver health remains underexplored. We assessed 30‐month changes in pulmonary, nutritional, metabolic and inflammatory markers in people with CF (PwCF) homozygous for F508del.

**Methods:**

We retrospectively analysed 112 PwCF (median age‐31 years) treated with ETI from July 2021 to December 2024. Clinical, spirometric and biochemical data were collected at baseline and at 6, 12, 24 and 30 months.

**Results:**

ETI produced sustained lung function gains (percent predicted FEV₁ + 15 points at 24 months, p < 0.001), BMI increase (+1.7 kg/m^2^ in year‐one, p < 0.001) and marked C‐reactive protein reduction (−80% at 6 months), with an 85% decrease in pulmonary exacerbations. Nutritional recovery shifted BMI distribution: underweight prevalence declined from 12.5% to 1.8%, while overweight rose from 15.2% to 27.7%. Adolescents improved in weight‐for‐age Z‐scores (+0.42, p = 0.01). Total and LDL cholesterol increased but remained within reference ranges; HDL, triglycerides and glycaemic control stayed stable, with no new cystic fibrosis–related diabetes (CFRD). Vitamin D improved; vitamin B12 fluctuated with supplementation. Mild, transient transaminase elevations occurred in 4.5% of PwCF, with no fibrosis progression (APRI/FIB‐4 below risk thresholds).

**Conclusion:**

ETI provides durable multisystem benefits, preserving lung function and improving nutritional and metabolic profiles. However, the shift towards overweight/obesity and biochemical signs of hepatic stress suggests evolving cardiometabolic risks. These findings support early ETI initiation and reinforce the need for ongoing monitoring of nutrition, lipid profile and liver function, together with updated CF care strategies to mitigate long‐term cardiometabolic complications.

**What is already known about this subject**

- Elexacaftor/tezacaftor/ivacaftor (ETI) has been shown to significantly improve lung function and nutritional status in patients with cystic fibrosis (CF).
- Long‐term real‐world evidence beyond 12 months remains scarce, particularly in paediatric and adolescent populations.
- The effects of ETI on metabolic, lipid and inflammatory profiles are still not well defined.

**What this study adds**

- This study represents a large and deeply phenotyped European real‐world cohort of paediatric and young adult CF patients homozygous for F508del treated with ETI, with longitudinal assessment of pulmonary, nutritional, inflammatory and metabolic outcomes.
- Demonstrates substantial reductions in systemic inflammation and early metabolic remodelling, including shifts in lipid parameters.
- Reveals heterogeneous weight and body composition trajectories based on baseline nutritional status, reinforcing the need for individualized dietary strategies in the modulator era.

## 1 BACKGROUND

Cystic fibrosis (CF) is a life‐limiting autosomal recessive disease caused by variants in the Cystic Fibrosis Transmembrane Conductance Regulator (CFTR) gene, leading to thick, dehydrated mucus that affects multiple organs, mainly the respiratory and gastrointestinal tracts.^2^, ^3^ Malabsorption and chronic inflammation contribute to malnutrition and fat‐soluble vitamin deficiencies.^4^ A distinct dyslipidaemia profile has been observed in PwCF, which may contribute to an elevated long‐term cardiovascular risk.^5^ Until recently, therapy for CF was mostly symptomatic. The triple CFTR modulator Elexacaftor/tezacaftor/ivacaftor (ETI) was approved in the US in 2019 and subsequently in the European Union in 2021 for PwCF with at least one *F508del* allele.

Phase III trials demonstrated that ETI can significantly improve lung function, nutritional status (BMI) and reduce pulmonary exacerbations (PEx).^6^ However, evidence regarding its long‐term impact on nutritional and biochemical parameters, including lipid profile, liver enzymes, glucose metabolism and fat‐soluble vitamins, remains limited. Although emerging real‐world studies have confirmed substantial benefits of ETI in broad CF populations, rigorous longitudinal analyses within homogeneous genotype cohorts are still lacking. We aimed to evaluate the 30‐month impact of ETI on pulmonary, nutritional and metabolic outcomes in a real‐world cohort of PwCF homozygous for *F508del (F508del/F508del)*, focusing on changes in BMI, metabolic parameters and the emerging risks of overweight and metabolic disease.

## 2 PATIENTS AND METHODS

### 2.1 Study design and data collection

We conducted a single‐centre retrospective cohort study at the Regional Reference CF Center of Policlinico Umberto I, Sapienza University of Rome. Eligible participants were PwCF aged >12 years, homozygous for the F508del mutation (confirmed by genetic sequencing), who initiated ETI therapy between July 2021 and December 2024. The study was approved by the Ethics Committee of Lazio 1 (Ref. 7609/CE Lazio1), and written informed consent was obtained from all participants or their legal guardians.

Exclusion criteria included the need for mechanical ventilation, CF‐related liver disease (cirrhosis), organ transplantation or pregnancy. Baseline characteristics and comorbidities (including CF‐related diabetes, pancreatic insufficiency, etc.) were recorded. CFRD was defined in accordance with the established guidelines, which require either an abnormal oral glucose tolerance test or fasting hyperglycaemia that necessitates insulin therapy.^7^

All patients received standard dietary counselling and pancreatic enzyme plus vitamin supplementation per CF guidelines.^8^ Clinical assessments were performed at baseline (T0) and at 6(T6), 12(T12), 24(T24) and 30(T30) months post‐ETI initiation. Pulmonary function was measured as percent predicted FEV₁ (ppFEV₁) by standard spirometry.^9^ Pulmonary exacerbations were defined as hospitalizations or intravenous antibiotic courses for worsening respiratory symptoms, and were recorded for 12 months before and after ETI initiation. Baseline lung function was defined by the last pre‐treatment spirometric value.

Nutritional status (weight, height, BMI) and laboratory parameters were evaluated at each visit. Biochemical panels included metabolic markers (fasting glucose, HbA₁c, total/LDL/HDL cholesterol, triglycerides), liver function tests (ALT, AST, alkaline phosphatase, GGT, bilirubin), inflammatory markers (C‐reactive protein, LDH) and vitamins (25‐OH vitamin D, vitamin B12). The aspartate aminotransferase‐to‐platelet ratio index (APRI) and the fibrosis‐4 index (FIB‐4) were also measured.^10^, ^11^ Vitamin D levels were categorized as normal (>30 ng/mL), insufficient (20–29.9) or deficient (<20), measured at the end of winter to minimize seasonal variation. All adverse events (AEs) documented in the patients' medical records during routine clinical visits throughout the study period were extracted and analysed retrospectively. Temporary discontinuation of ETI was implemented in cases where hepatic transaminases exceeded five times the upper limit of normal (ULN), in accordance with standard safety protocols. Data completeness exceeded 95% for all primary endpoints across time points. Missing data were primarily due to loss to follow‐up or incomplete laboratory panels and were handled via pairwise deletion in the analyses.

### 2.2 Statistical analysis

Data analysis was performed using RStudio (v4.3.0), Jamovi (v2.4) and Python (v3.11). Continuous variables were expressed as mean ± SD or median with interquartile range (IQR), and categorical data as absolute and relative frequencies. Normality was assessed via Shapiro–Wilk test and graphical inspection. Longitudinal changes were analysed using the Friedman test with Durbin‐Conover post‐hoc comparisons. Between‐group differences (ETI‐naïve *vs*. pretreated) were assessed using Mann–Whitney U tests for continuous variables and χ^2^ or Fisher's exact tests for categorical variables. Spearman's correlation evaluated associations between baseline values and clinical changes. Multivariable linear regression models were used to explore predictors of treatment response. A p‐value <0.05 was considered statistically significant.

### 2.3 Nomenclature of targets and ligands

Key protein targets and ligands in this article are hyperlinked to corresponding entries in http://www.guidetopharmacology.org, and are permanently archived in the Concise Guide to PHARMACOLOGY 2021/22.

## 3 RESULTS

### 3.1 Cohort characteristics

We enrolled 112 *F508del/F508del* patients (49.1% female, median age 31 years, range 12–76). Baseline characteristics are summarized in Table 1. At baseline, they had moderate lung disease (mean ppFEV₁ = 71.2 ± 26.3) and near‐normal nutritional status (mean BMI 21.7 ± 3.8 kg/m^2^). Of these, 29 PwCF (25.9%) had been on lumacaftor/ivacaftor (LI, Orkambi) for≥6 months prior to ETI, while 83 (74.1%) were modulator‐naïve. All PwCF completed the 12‐month follow‐up after ETI initiation; however, only 100 patients (89.3%) and 93 (83.0%) were followed up to 24 and 30 months from baseline, respectively.

**TABLE 1:** Baseline demographic and clinical characteristics of the study population (N = 112), including sex distribution, age, CFTR modulator history, ppFEV₁ and BMI at enrollment.

Baseline characteristics	
Characteristics	N = 112
Female sex ‐ n (%)	55 (49.1%)
Age in years ‐ mean (SD)	31.01 (±14.88)
**CFTR modulator history‐ n (%)**	
Naive	83 (74.10%)
Lumacaftor + Ivacaftor	29 (25.90%)
ppFEV1 ‐ mean (IQR)	71.2 ± 26.3
BMI ‐ mean (IQR)	21.7 ± 3.8 kg/m^2^
**PEx ‐ number of patients**	47
*Number of events*	*72*
▪ Patients with 1 event	28
▪ Patients with 2 events	14
▪ Patients with 3 events	4
▪ Patients with 4 events	1

### 3.2 Pulmonary outcomes

**FIGURE 1:**
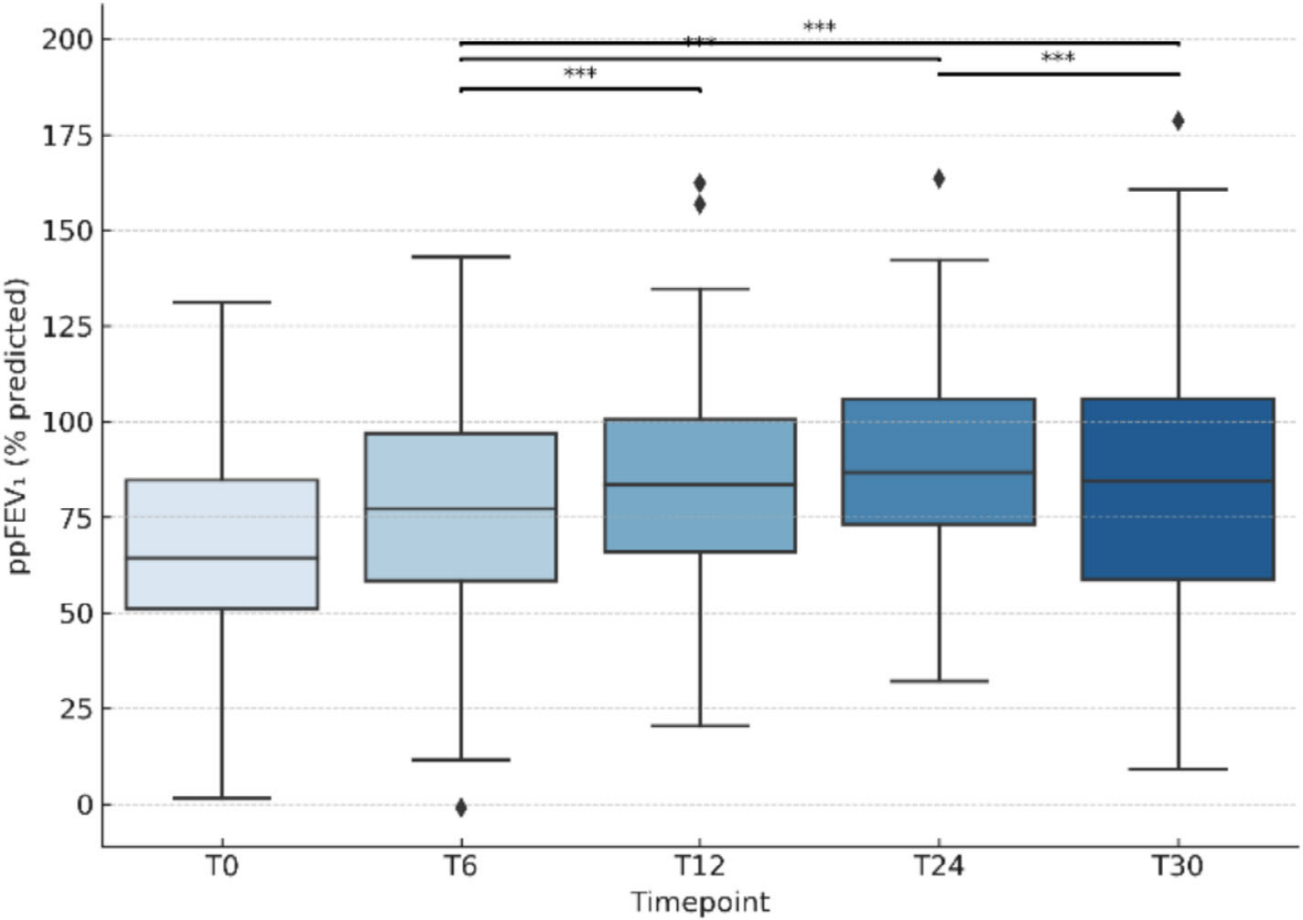
**Longitudinal distribution of percent predicted FEV₁ (ppFEV₁) over 30 months.** The figure illustrates ppFEV₁ values at baseline (T0) and at 6‐, 12‐, 24‐ and 30‐months follow‐up visits after initiation of elexacaftor/tezacaftor/ivacaftor (ETI) in individuals with cystic fibrosis. Significant improvements were observed at all follow‐up time points, with gains sustained throughout the 30‐month period. Asterisks indicate statistically significant differences based on one‐way ANOVA with Tukey's post hoc test (*p < 0.05, **p < 0.01, ***p < 0.001).

ETI led to rapid and sustained lung function improvement. Figure 1 illustrates the ppFEV₁ trajectory over 30 months of ETI. Within 6 months, mean ppFEV₁% increased from 71% at baseline to 81% (+10 points; *p* < 0.001). By 12 months, ppFEV₁ further rose to 84% on average (+13 points *vs*. baseline; *p* < 0.001). This improvement plateaued after the first year: at 24 months, mean ppFEV₁ was 86% (+15 points from baseline), and it remained stably elevated around the mid‐80s through 30 months (no significant decline from the 1‐year peak). PEx markedly decreased following ETI initiation. In the year prior to treatment, 42% of PwCF experienced ≥1 PEx (72 total events), compared to only 7% (9 events) in the first year on ETI. This corresponds to 85% fewer patients with exacerbations and 88% with fewer acute events.

### 3.3 Nutritional and metabolic outcomes

**FIGURE 2:**
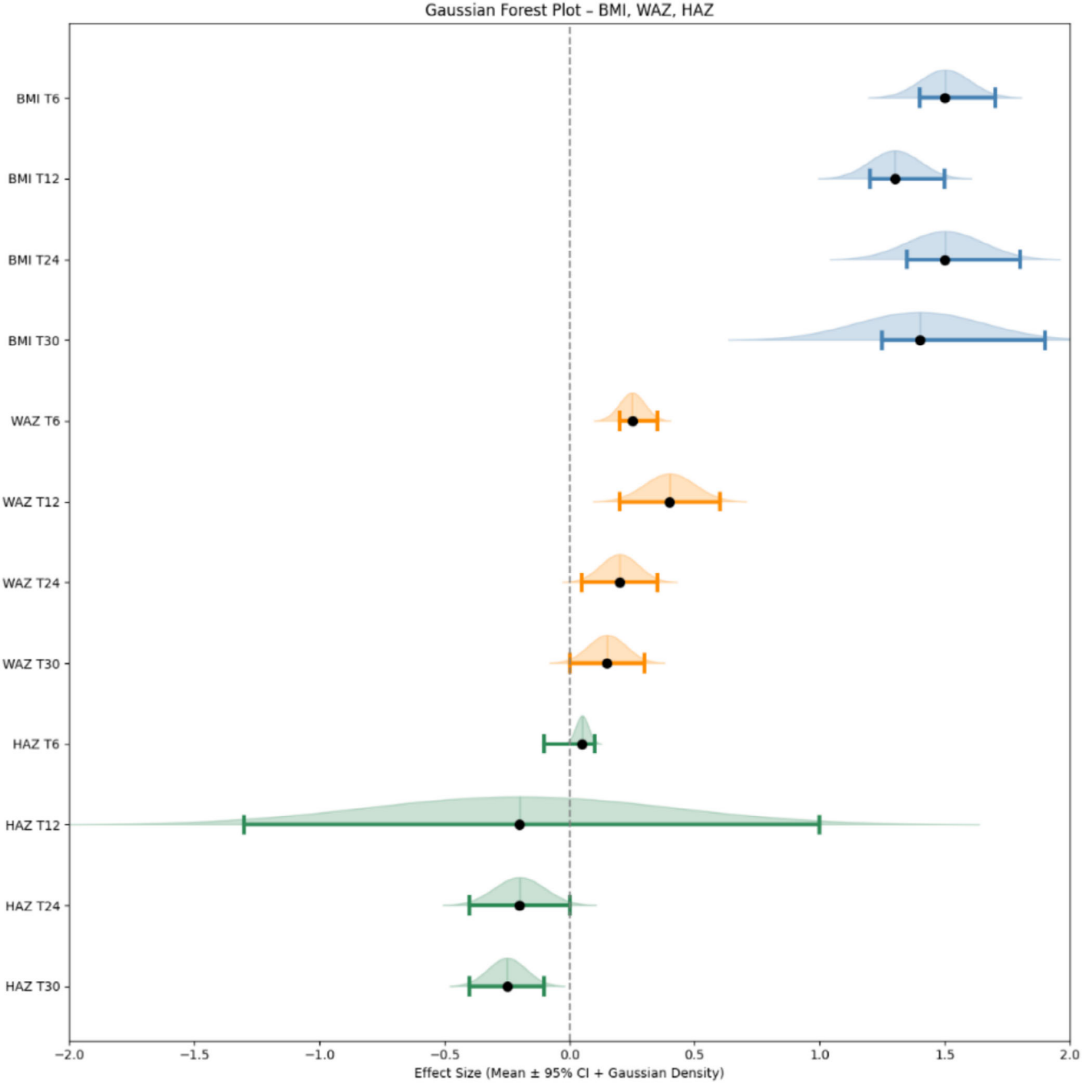
**Changes in BMI, weight‐for‐age (WAZ) and height‐for‐age (HAZ) Z‐scores during ETI therapy in adolescents with cystic fibrosis.** Gaussian forest plot showing mean changes from baseline (T0) with 95% confidence intervals at 6, 12, 24 and 30 months of ETI therapy. Each data point represents the mean effect size, with horizontal bars denoting 95% confidence intervals. The Gaussian density is indicative of the distribution of error around each estimate. The vertical dashed line denotes the absence of deviation from the baseline. BMI increased significantly during the first year and remained stable thereafter. WAZ improved in the first 12 months, while HAZ changes were modest and not statistically significant. All values are expressed as mean difference from baseline. Data for WAZ and HAZ were derived from the adolescent subgroup (12–18 years; n = 26).

Nutritional status improved significantly during ETI therapy. Baseline BMI increased from 21.7 ± 3.8 to 23.4 ± 4.2 kg/m^2^ in the first year (Δ + 1.7 kg/m^2^, p < 0.001; Figure 2), with rapid gains in the first 12 months followed by stabilization at a higher set point (22.5 ± 2.7–23.2 ± 4.3 kg/m^2^ at 24–30 months; p < 0.001 *vs*. baseline). Nearly all PwCF gained weight, and 88% of those who were underweight (BMI < 18.5) at baseline achieved a normal BMI. A significant inverse correlation was observed between baseline BMI and percent weight gain (ρ ≈ −0.40, p < 0.001), indicating that PwCF with poorer nutritional status derived the greatest benefit. Over 30 months, underweight prevalence declined from 14 PwCF (12.5%) to 2 (1.8%), whereas overweight increased from 17 (15.2%) to 31 (27.7%), reflecting a rightward shift in BMI distribution. In the adolescent subgroup (12–18 years, n = 26), weight‐for‐age Z‐scores (WAZ) improved significantly in the first year (Δ + 0.42 at 12 months, p = 0.01) and remained stable thereafter, while height‐for‐age Z‐scores (HAZ) showed modest, nonsignificant increases (Δ + 0.18 at 30 months, p = 0.12). Taken together, these findings suggest that ETI may support catch‐up growth in weight among adolescents, while having no significant effect on linear growth over time (Figure 2).

Metabolic parameters also changed (Table 2). Total cholesterol rose from 3.77 ± 0.94 to 4.30 ± 0.98 mmol/L (145.6 to 166 mg/dL, p < 0.001) and LDL from 1.72 ± 0.75 to 2.52 ± 0.98 mmol/L (66.4 to 90.3 mg/dL, p < 0.01), while HDL remained stable (median 1.51 mmol/L) and triglycerides did not change (median 0.83 mmol/L). Despite statistical significance, lipid values remained within reference ranges, consistent with improved absorption and nutritional recovery rather than dyslipidaemia.

**TABLE 2:** Longitudinal changes in nutritional, inflammatory and hepatic parameters in PwCF treated with elexacaftor/tezacaftor/ivacaftor (ETI) over a 30‐month follow‐up. Values are expressed as mean or median (±SD), and corresponding p‐values refer to comparisons with baseline (T0).

Parameter	Statistic	T0	T6	T12	T24	T30
***Nutritional profile***						
**Total Cholesterol (mmol/L)**	Mean (SD)	3.77 ± 0.94	4.19 ± 0.82	4.25 ± 0.88	4.27 ± 0.99	4.30 ± 0.98
	p‐value	‐	**0.033**	**0.005**	**0.003**	**<0.001**
**LDL (mmol/L)**	Median (SD)	1.72 ± 0.75	2.27 ± 6.52	2.12 ± 1.12	2.18 ± 0.90	2.52 ± 0.98
	p‐value	‐	**0.015**	0.87	**0.028**	**<0.001**
**HDL (mmol/L)**	Mean (SD)	1.41 ± 0.37	1.58 ± 0.34	1.44 ± 0.35	1.51 ± 0.30	1.59 ± 0.47
	p‐value	‐	0.265	0.961	0.147	0.593
**Triglycerides (mmol/L)**	Median (SD)	0.78 ± 0.41	0.85 ± 0.40	0.89 ± 0.38	0.81 ± 0.46	0.83 ± 0.47
	p‐value	‐	0.571	0.323	0.396	0.371
**Vitamin D (ng/mL)**	Mean (SD)	21.40 ± 8.78	24.30 ± 8.62	24.10 ± 10.49	27.90 ± 11.53	25.30 ± 11.53
	p‐value	‐	0.640	0.191	0.126	0.191
**Vitamin B12 (pg/mL)**	Median (SD)	812.00 ± 302.07	628.00 ± 310.28	668.00 ± 299.31	740.00 ± 343.99	767.00 ± 369.44
	p‐value	‐	0.087	**0.046**	0.389	0.502
**Glucose (mmol/L)**	Mean (SD)	5.21 ± 1.05	5.03 ± 1.00	5.21 ± 1.39	5.28 ± 1.15	5.22 ± 1.16
	p‐value	‐	**0.045**	0.509	0.813	0.706
**HbA**_**1**_ **c (%)**	Mean (SD)	5.91 ± 0.75	5.71 ± 0.69	5.56 ± 0.6	5.69 ± 0.68	5.71 ± 0.75
	p‐value	‐	0.332	0.798	0.645	0.720
***Inflammation and liver function***						
**CRP (mg/L)**	Mean (SD)	10.30 ± 12.37	2.00 ± 8.96	3.75 ± 10.40	3.37 ± 10.48	3.05 ± 3.73
	p‐value	‐	**<0.001**	**0.002**	**0.014**	**<0.001**
**AST (U/L)**	Mean (SD)	19.70 ± 8.94	25.30 ± 9.15	29.70 ± 19.44	21.50 ± 7.48	21.40 ± 6.62
	p‐value	‐	**0.006**	**0.003**	0.083	0.671
**ALT (U/L)**	Mean (SD)	20.10 ± 11.79	30.50 ± 18.11	33.00 ± 25.22	26.70 ± 14.20	24.60 ± 10.30
	p‐value	‐	**<0.001**	**0.002**	**0.020**	0.077
**GGT (U/L)**	Mean (SD)	14.70 ± 11.40	16.90 ± 8.96	18.80 ± 16.64	17.00 ± 13.33	14.80 ± 9.63
	p‐value	‐	**0.020**	**0.028**	0.129	0.663
**Alkaline Phosphatase (U/L)**	Mean (SD)	114.00 ± 87.25	134.00 ± 97.96	127.00 ± 66.68	117.00 ± 43.58	111.00 ± 37.63
	p‐value	‐	0.078	0.263	0.640	0.888
**Total Bilirubin (mg/dL)**	Mean (SD)	0.33 ± 0.28	0.70 ± 0.36	0.69 ± 0.51	0.79 ± 0.66	0.71 ± 0.55
	p‐value	‐	**<0.001**	**<0.001**	**<0.001**	**<0.001**
**Conjugated Bilirubin (mg/dL)**	Mean (SD)	0.17 ± 0.12	0.29 ± 0.12	0.31 ± 0.20	0.30 ± 0.20	0.30 ± 0.19
	p‐value	‐	**<0.001**	**<0.001**	**<0.001**	**<0.001**

Fat‐soluble vitamins showed favourable trends. Mean 25‐OH vitamin D rose from 21.4 ± 8.8 ng/mL to 27.9 ± 11.5 ng/mL at 24 months, though not statistically significant (Table 2). Vitamin B12, often elevated due to supplementation, declined transiently at 12 months (median 812 to 668 pg/mL, Δ − 18%; p = 0.046) before returning near baseline by 30 months, likely reflecting adjustments in supplementation as malabsorption improved.

Glycaemic control remained stable throughout follow‐up: fasting glucose (baseline 5.21 ± 1.05 *vs*. 5.22 ± 1.16 mmol/L at 30 months) and HbA₁c (5.91 ± 0.75% *vs*. 5.71 ± 0.75%) did not change significantly (Table 2). Thirteen PwCF (11.6%) had established CFRD at baseline, all of whom remained insulin‐dependent. No new cases occurred over 30 months. Within this subgroup, some reported reduced insulin requirements, and three described episodes consistent with reactive hypoglycaemia.

### 3.4 Inflammation and liver function

Systemic inflammation was significantly reduced under ETI. The median CRP level fell from 10.3 ± 12.37 mg/L at baseline to 2.0 ± 8.96 mg/L after six months (∆ = −80%, p < 0.001), remaining low at 12 months (3.75 ± 10.40 mg/L, p = 0.002). A slight increase in CRP was observed by 30 months (3.05 ± 3.73 mg/L), which was still significantly below baseline (p < 0.001). However, no PwCF experienced a severe inflammatory exacerbation during ETI, reinforcing the modulators' potent effect in reducing chronic inflammation.

There was no significant correlation between CRP reduction and ppFEV₁ or BMI improvements (ρ ≈ −0.21 for ΔCRP *vs*. ΔFEV₁), suggesting that the anti‐inflammatory effect of ETI may occur independently of individual lung function gains.

**FIGURE 3:**
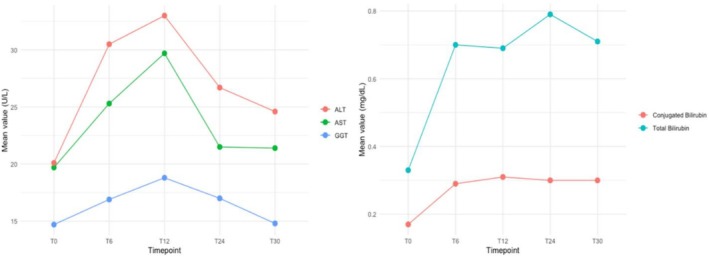
**Longitudinal trends in hepatic biomarkers following ETI initiation.** Mean serum levels of alanine aminotransferase (ALT), aspartate aminotransferase (AST), total bilirubin and direct bilirubin measured at baseline (T0) and at 6, 12, 24 and 30 months after starting elexacaftor/tezacaftor/ivacaftor (ETI) therapy. As demonstrated in Figure 3A (left), transient rise in transaminases was observed during the first year, peaking at 12 months, followed by a decline towards baseline levels by month 30. Both ALT and AST remained within normal ranges throughout. GGT levels remained stable throughout the follow‐up period, with no significant fluctuations. Total and direct bilirubin levels (Figure 3B ‐ right) showed a mild but statistically significant increase post‐treatment, yet remained clinically acceptable. These trends support the hepatic safety profile of ETI, with no evidence of sustained hepatotoxicity over long‐term follow‐up.

Liver function remained generally stable throughout the 30‐month observation period, with only transient, low‐grade enzyme elevations occurring during the first year of ETI (Figure 3). The mean ALT increased from 20.1 ± 11.79 U/L at baseline to 30.5 ± 18.11 U/L at 6 months (p < 0.001), while mean AST rose from 19.7 ± 8.94 U/L to 25.3 ± 9.15 U/L (p = 0.006). Peak values were observed at 12 months (ALT 33.0 U/L, p = 0.002; AST 29.7 U/L, p = 0.003), followed by a progressive return towards baseline by 30 months (ALT 24.6, AST 21.4 U/L; no significant difference *vs*. T0). Transaminase levels remained within normal ranges in most individuals at all time points.

Five PwCF (4.5%) experienced transient ALT and/or AST elevations exceeding 3 × the upper limit of normal (ULN) during the first 3–6 months of treatment. Among these, three continued ETI without interruption, while two underwent short precautionary treatment holds followed by successful re‐challenge. No participants showed clinical signs or biochemical patterns consistent with drug‐induced hepatitis or hepatic decompensation. None exceeded 5 × ULN.

Baseline mean GGT level increased progressively from 14.7 ± 11.40 U/L to 18.8 ± 16.64 after one year (p = 0.028). Alkaline phosphatase levels showed no significant change. Total bilirubin increased from 0.33 ± 0.28 to 0.71 ± 0.55 mg/dL over 30 months (p < 0.001), and direct bilirubin from 0.17 ± 0.12 to 0.30 ± 0.19 mg/dL (p < 0.001), with all values remaining within the clinically acceptable range (Figure 3). To assess hepatic fibrosis risk, APRI [APRI = 100*(AST/AST upper limit of normal [ULNAST]) ÷ platelet count (109/L)] and FIB‐4 [FIB‐4 = (Age x AST) / (Platelet count x √ALT)] indices were calculated at the cohort level and for individuals with transaminase elevations >3 × ULN (Supplementary Table 1).

In the overall cohort, all APRI values remained below 0.5 and all FIB‐4 values below 0.7, indicating minimal to no risk of significant fibrosis. In the subgroup of five individuals with transaminase elevations, peak APRI and FIB‐4 scores were 1.18 and 1.37, respectively, both below the fibrosis thresholds recommended in CF‐specific guidelines (APRI <1.5; FIB‐4 < 2.0 for adults under 65 years).^12^ These values support the absence of clinically significant or advanced fibrosis even among patients with transient enzyme abnormalities.

### 3.5 Subgroup analysis

Among 112 participants, 29 (25.9%) had prior exposure to lumacaftor/ivacaftor (LI), while 83 (74.1%) were modulator‐naïve. Both subgroups exhibited substantial improvements following ETI initiation (Supplementary Table, S2). Despite lower baseline ppFEV₁ in LI‐treated patients (66.4% *vs*. 73.1%, *p* < 0.01), both groups achieved significant ppFEV₁ improvements over time, with mean increases of approximately +15% from baseline (Δ + 14.8% in the LI group *vs*. + 15.3% in the naïve group), reaching 81.2% and 88.4%, respectively. Weight gain was observed in both cohorts, although more pronounced in naïve patients at 12 months (+1.5 *vs*. + 0.8 kg/m^2^, *p* = 0.04). By 24 months, BMI converged around normal values in both groups. Exacerbation rates declined by approximately 70% across both subgroups, closely aligning with the 85% reduction observed in the overall population, thereby supporting consistent clinical benefit regardless of prior modulator exposure. Adolescents (12–18 years) and adults (>18 years) both exhibited sustained ppFEV₁ improvements following ETI initiation. Baseline ppFEV₁ was higher in adolescents (91.1% *vs*. 64.8%, p < 0.001), and the differential remained significant throughout follow‐up. At 30 months, mean ppFEV₁ increased to 117.5% in adolescents and 75.2% in adults (p < 0.001), reflecting robust pulmonary gains across age groups, with greater absolute lung function preservation in younger individuals (Table 3).

**TABLE 3:** Longitudinal changes in percent predicted FEV₁ (ppFEV₁) following ETI initiation, stratified by age group (12–18 years *vs.* >18 years). At all time points, adolescents showed significantly higher ppFEV₁ values compared to adults (p < 0.001), with both groups demonstrating sustained improvement over a 30‐month period of follow‐up.

Parameter		Comparison by age	T0	T6	T12	T24	T30
**ppFEV**_**1**_		**12–18** Mean (SD)	91.1 ± 20.2	102.1 ± 20.7	103.8 ± 19.9	108.1 ± 20.3	117.5 ± 24.0
		**>18** Mean (SD)	64.8 ± 24.9	75.0 ± 26.4	78.2 ± 26.3	79.5 ± 23.6	75.2 ± 29.3
		**p‐value**	**<0.001**	**<0.001**	**<0.001**	**<0.001**	**<0.001**

## 4 DISCUSSION

Our 30‐month real‐world study demonstrates that ETI provides sustained and clinically meaningful benefits in PwCF homozygous for F508del. Improvements were evident in pulmonary function, nutritional status and systemic inflammation. The mean ppFEV₁ gain of approximately 10–15 percentage points mirrors results from pivotal 24‐week trials and real‐world cohorts^6^, ^13^, ^14^ and aligns with our current findings in a paediatric population.^15^ The significant reduction in PEx (∆ = −85%) indicates a paradigm shift in the management of CF, with many PwCF transitioning from frequent exacerbations to a new baseline of clinical stability, characterized by preserved lung function reserve and infrequent acute events.^16^, ^17^ These benefits were durable, effectively halting the progressive decline of the pre‐modulator era.^9^

Nutritional improvements were notable. Achieving a BMI in the 22–23 kg/m^2^ range, as observed in most adults on ETI, represents a positive prognostic marker, reflecting improved energy reserves. In adolescents, weight‐for‐age Z‐scores improved significantly, whereas height‐for‐age Z‐scores showed only modest, nonsignificant changes. This pattern suggests that ETI may preferentially support weight recovery and nutritional reserves during adolescence, consistent with BMI gains in adults. The lack of height catch‐up may reflect limited growth potential in older adolescents or highlight the importance of earlier modulator initiation to fully influence linear growth. These findings align with registry reports indicating that weight recovery typically precedes improvements in stature, reinforcing the need for timely therapy to optimize growth trajectories.^18^, ^19^ Weight trajectories differed according to baseline status. Underweight PwCF experienced rapid catch‐up growth (often >15% bodyweight in the first year), whereas overweight individuals paradoxically showed modest BMI reductions (−1.3 kg/m^2^), bringing them closer to the normal range. Although we did not directly assess body composition in our cohort, previous CT‐based analyses have shown that ETI treatment in adults with CF leads to significant increases in both fat and lean mass, with fat mass representing the predominant component of weight gain.^20^ In addition, paediatric data suggest a trend towards increased adiposity and hepatic lipid accumulation, as reflected by rising BMI and serum bilirubin levels during ETI therapy, despite stable transaminases and the absence of overt liver dysfunction.^21^

Accordingly, the modest BMI reductions observed in overweight individuals in our cohort may reflect heterogeneity in treatment response, improved nutrient absorption or reduced systemic inflammation, rather than selective lean mass gain. Importantly, systemic inflammation declined significantly, as evidenced by more than 80% average reduction in CRP levels, suggesting a less catabolic and more metabolically efficient state, which may favour overall nutritional recovery without necessarily altering body composition in a targeted manner. These findings are consistent with recent paediatric studies, where significant reductions in IgG, IgA, leukocyte counts and platelet levels were documented following ETI initiation, further supporting the hypothesis that ETI attenuates systemic inflammation and promotes immunometabolic stabilization.^22^

Collectively, these findings indicate that while ETI supports recovery from undernutrition in PwCF, it may also promote increases in adiposity in a subset of patients, particularly those with favourable baseline nutritional status. This emphasizes the necessity to transform conventional high‐fat, high‐calorie CF diets, which were initially developed to prevent wasting, into personalized approaches that balance nutritional rehabilitation with long‐term metabolic health. As overweight and obesity levels rise in the modulator era, from approximately 15% pre‐ETI to nearly 40% in the modulator era, there is an increasing need to monitor not just weight, but also body composition and cardiometabolic risk over time.^1^, ^23^ This need is further reinforced by emerging evidence of ETI‐induced metabolic remodelling, including elevations in BMI, total cholesterol and LDL even in paediatric cohorts, suggesting early shifts towards a more atherogenic profile.^24^ Our cohort reflected this trend, with underweight prevalence declining from 14 patients (12.5%) to 2 (1.8%) over 30 months, while overweight prevalence rose from 17 (15.2%) to 31 (27.7%). Although higher BMI has long been associated with improved survival in CF, excessive adiposity may introduce conventional cardiometabolic risks, including hypertension and metabolic syndrome. Accordingly, nutritional guidelines should evolve from maximizing caloric intake towards strategies that emphasize balance and metabolic health.^25^

Alongside clinical improvements, ETI appears to reveal emerging metabolic phenotypes in PwCF.^26^ While PwCF became clinically more stable and more anabolic, they also began to display metabolic profiles more akin to the general population. Total cholesterol and LDL levels increased by approximately 10–15% on ETI (reaching mid‐normal levels), while triglyceride levels remained stable. These shifts likely reflect enhanced intestinal fat absorption and attenuated systemic inflammation, contributing to a reversal of the ‘CF‐typical’ hypocholesterolaemia seen despite historically high dietary fat intake.

Although lipid levels remained within reference values, the upward trend in cholesterol and BMI raise concerns about a potential predisposition to cardiometabolic complications in the modulator era. Recent paediatric data support this interpretation: a longitudinal biochemical analysis revealed significant increases in total cholesterol, LDL and liver‐associated markers after 12 months of ETI therapy in adolescents homozygous for F508del, even in the absence of overt hepatic dysfunction.^27^ These early metabolic shifts may represent the initial stages of a broader transition towards phenotypes previously uncharacteristic of CF. This highlights the need to re‐evaluate nutritional strategies and to incorporate cardiovascular risk assessment into the standard care of CF.^28^

Mild, transient elevations in ALT and AST were observed during the first year of ETI, with median ALT increasing from 19 to 22 U/L, consistent with findings from pivotal clinical trials. These increases were more pronounced among PwCF with a higher baseline BMI (≥22 kg/m^2^) or those who experienced rapid weight gain, suggesting that hepatic overload may contribute to subclinical steatosis. Emerging reports describe metabolic dysfunction‐associated steatotic liver disease (MASLD) in PwCF who attain normal weight or overweight on ETI, a scenario historically uncommon in CF but now plausible given improved weight and longevity.^29^

We did not formally diagnose MASLD in our cohort; no PwCF underwent imaging or biopsy for steatosis during the 30‐month observation. Thus, the *emergence of MASLD* reflects a potential risk inferred from weight gain and biochemical trends rather than confirmed cases. Nonetheless, the increases in BMI and cholesterol observed in some patients warrant vigilance for MASLD as survival improves.

No PwCF developed evidence of liver fibrosis or CFLD. Routine elastography or liver imaging was not performed, which represents a limitation. However, transaminase values remained within or close to the normal range, and synthetic function (as reflected by stable serum albumin) was preserved. Application of noninvasive fibrosis indices (APRI and FIB‐4) further supports the interpretation that the enzyme elevations were transient and benign, consistent with recent recommendations for CFLD screening.^12^ Consistent with prior studies, we observed a modest but statistically significant increase in both total and direct bilirubin levels during ETI treatment, while remaining within the clinical reference range. Although the precise aetiology of this trend is unclear, potential explanations include modulation of hepatic transport mechanisms, such as CFTR‐related changes in bile acid and organic anion transporter activity, rather than direct hepatocellular toxicity. Further mechanistic studies are needed to clarify these findings.

Although our findings indicate no evidence of progressive liver injury over 2.5 years, the question remains whether adjunctive interventions, such as dietary modification or pharmacotherapy, may mitigate hepatic effects in PwCF who develop features of MASLD or metabolic syndrome. Preclinical studies have suggested potential benefit from insulin sensitizers or GLP‐1/SGLT2 inhibitors, and in a CF rabbit model, the SGLT1/2 inhibitor sotagliflozin reduced steatosis and improved metabolic outcomes during ETI.^30^ These data underscore that improving cardiometabolic health is becoming a new frontier in CF care, as the clinical focus shifts from preventing CF‐specific organ failure to managing comorbidities more typical of ageing populations.^31^, ^32^

Emerging data suggest that endocrine pancreatic function may improve under ETI. CFRD remains a significant complication in older patients, with a historical prevalence of more than 50% among adults with CF.^33^ The early initiation of highly effective modulators has been proposed as a strategy to delay or prevent its onset by enhancing insulin secretion and reducing inflammation.^34^

In our cohort, fasting glucose and HbA₁c remained stable over 30 months, with no new cases of CFRD. All PwCF with pre‐existing CFRD remained insulin‐dependent, but some reported reduced insulin requirements, and three experienced episodes consistent with reactive hypoglycaemia. These findings suggest that ETI may enhance insulin sensitivity or partially relieve β‐cell stress. Similar trends have been documented in multicentre studies, where mean insulin requirements declined significantly (0.85 to 0.71 U/kg/day after one year, p < 0.001) and up to 30% of PwCF regressed from CFRD to pre‐diabetes.^35^ Although our study did not include formal glucose tolerance testing, the alignment between stable laboratory indices and patient‐reported improvements supports the hypothesis that ETI contributes to stabilization or partial recovery of endocrine pancreatic function. HbA₁c changes were modest, indicating that CFRD is not fully reversible, yet improved β‐cell activity appears evident. The occurrence of reactive hypoglycaemia underscores the need for individualized monitoring and therapy adjustment. Collectively, these observations reinforce the view that ETI is reshaping the endocrine phenotype of CF and may lessen the long‐term burden of CFRD and its complications.^36^

PwCF showed a deficit in the absorption of fat‐soluble vitamins, especially vitamin D, which plays a crucial role in bone development and immune system modulation.^37^ In our study, we observed an increase in vitamin D levels, although this was not statistically significant. This trend may be attributable to a reduction in intestinal inflammation and the subsequent enhancement of absorption, which is consistent with the findings of several other studies.^38^, ^39^

At the cellular level, our findings align with emerging research that CFTR modulators have far‐reaching effects on cell metabolism and immunity. CF epithelial and immune cells inherently exhibit a hypermetabolic, pro‐inflammatory state due to chronic stress. ETI appears to reprogram cellular energy balance. A recent study showed that triple therapy normalizes mitochondrial respiration in CF cells by restoring calcium homeostasis, thereby improving efficiency and reducing oxidative damage.^40^ Macrophage function also improves: macrophages from PwCF treated with ETI demonstrated more normalized metabolism and improved bacterial phagocytosis.^41^ These mechanistic insights help to explain the clinical reductions in inflammation and infections that we observed. By partially correcting the cellular defects underlying CF (not only ion transport, but also immune cell dysfunction), ETI creates a ripple effect of reduced inflammation, improved pathogen clearance and enhanced organ function. These pleiotropic benefits demonstrate why highly effective modulators are transformative; they do not simply raise FEV₁, but also shift the body from a catabolic, inflamed state to a healthier, anabolic state.

The real‐world data presented here confirm the favourable safety profile of ETI over a 30‐month period. Apart from transient liver enzyme elevations (<5% of PwCF, managed with monitoring or brief interruption), no significant adverse events attributable to ETI were observed. No renal or haematological toxicities were observed. Only one patient required a temporary discontinuation due to a rash, which resolved with topical steroids, and ETI was successfully rechallenge without recurrence. This level of tolerability mirrors clinical trial results and growing post‐marketing evidence. Guidelines now recommend routine liver function tests during the first 6–12 months (monthly to quarterly), a protocol we followed to promptly detect asymptomatic ALT elevations.^42^ All our patients were able to continue ETI long‐term, with none discontinuing due to side effects. This high adherence highlights that the benefits vastly outweighed any mild adverse effects.

Previous use of older modulators, such as LI, did not reduce the effectiveness of ETI. Even PwCF with advanced disease achieved significant improvements, many reaching lung function levels never attained on LI. By the end of the study, both the naïve and pre‐treated groups showed comparable outcomes in terms of nutrition and exacerbation rates, reinforcing the superiority of ETI and supporting its widespread adoption. These findings highlight that, while early initiation yields optimal results, significant benefits can be achievable even in late‐stage patients, thus supporting the current consensus for early, universal ETI treatment in CF.

The limitations of this study include its single‐centre, retrospective design and lack of a control group. While the improvements observed are most likely attributable to ETI, we cannot fully exclude the possibility of contributions from improved adherence to other therapies due to the boost in health and optimism resulting from ETI. Follow‐up beyond 24 months included fewer patients due to late ETI initiation or loss to follow‐up, so long‐term trends should be interpreted with caution. Furthermore, we did not directly quantify certain outcomes of interest, such as changes in sputum microbiology or exocrine pancreatic function (e.g. faecal elastase levels), which could have provided objective evidence of improved CFTR function in the lungs and gut.

## 5 CONCLUSIONS

This 30‐month real‐world study confirms that ETI therapy produces sustained, multi‐system benefits in PwCF homozygous for *F508del*. ETI significantly improved pulmonary function and nutritional status, drastically reducing pulmonary exacerbations and inflammation, while being associated with an optimal safety and tolerability profile. These results highlight the paradigm shift ETI has introduced in CF care, transforming a formerly relentless disease into one where long‐term stability and even the regression of certain complications are achievable. However, our findings underscore the importance of vigilance regarding new challenges in the ‘post‐modulator era’, such as obesity, metabolic syndrome and MASLD, as the CF population's health profile increasingly resembles that of the general population. Guidelines for CF care should be updated to address this evolving landscape by integrating nutritional monitoring and metabolic screening into routine management. Overall, our findings strongly support the early and universal adoption of ETI, as it mitigates classic CF manifestations and redefines the disease's clinical evolution in the modulator era.

## AUTHOR CONTRIBUTIONS

The authors confirm that the PI for this paper is Giuseppe Cimino and that he had direct clinical responsibility for patients.

Design: Nicola Perrotta, Luigi Angelo Fiorito, Giuseppe Cimino. Conduct/data collection/interpretation: Giulia Amato, Roberta Vescovo, Anna Virgilio. Analysis: Nicola Perrotta, Roberta Vescovo. Writing manuscript: Nicola Perrotta, Luigi Angelo Fiorito.

## CONFLICT OF INTEREST STATEMENT

All authors report no conflicts of interest relevant to this article.

## Supporting information


**Table S1.** **Non‐invasive Fibrosis Indices (APRI and FIB‐4) in the overall cohort and in the subgroup with ALT/AST elevations.** APRI and FIB‐4 values were calculated from cohort‐level mean AST and ALT at each timepoint (baseline, 6, 12, 24 and 30 months) using standard formulas. The upper limit of normal (ULN) for AST was set at 40 U/L, and the mean cohort age was 31 years. APRI was calculated as (AST/ULN_AST × 100) / Platelets (10⁹/L), and FIB‐4 as (Age × AST) / (Platelets × √ALT). All APRI values in the overall cohort remained below the threshold of 0.5 used to rule out advanced fibrosis.^1^ Similarly, all FIB‐4 scores were far below the standard threshold of 1.45 used in clinical practice to exclude significant liver fibrosis.^1^ The subgroup of five participants with ALT/AST elevations >3 × ULN also demonstrated low fibrosis indices, consistent with the absence of clinically significant or advanced liver fibrosis even among those with transient enzyme elevations.
**Table S2.** Pulmonary and nutritional response to ETI in patients previously treated with lumacaftor/ivacaftor (LI) *vs.* modulator‐naïve individuals. Although the LI group started from a lower baseline lung function, both subgroups experienced comparable improvements in ppFEV₁ and BMI, with no significant differences in exacerbation reduction.

## Data Availability

The data that support the findings of this study are available on request from the corresponding author and are not publicly available due to privacy or ethical restrictions.
