# Supplementary material for: Long‐term impact of Elexacaftor/Tezacaftor/ivacaftor on pulmonary, nutritional and metabolic outcomes in homozygous F508del cystic fibrosis patients: A real‐world cohort study
Source: Br J Clin Pharmacol. 2026 Jan 23;92(6):1822–32. doi: 10.1002/bcp.70460 (PMC13206182; doi:10.1002/bcp.70460)
Supplement: Supplementary file 1 — Table S1. Non‐invasive Fibrosis Indices (APRI and FIB‐4) in the overall cohort and in the subgroup with ALT/AST elevations. APRI and FIB‐4 values were calculated from cohort‐level mean AST and ALT at each timepoint (baseline, 6, 12, 24 and 30 months) using standard formulas. The upper limit of normal (ULN) for AST was set at 40 U/L, and the mean cohort age was 31 years. APRI was calculated as (AST/ULN_AST × 100) / Platelets (10⁹/L), and FIB‐4 as (Age × AST) / (Platelets × √ALT). All APRI values in the overall cohort remained below the threshold of 0.5 used to rule out advanced fibrosis. 1 Similarly, all FIB‐4 scores were far below the standard threshold of 1.45 used in clinical practice to exclude significant liver fibrosis. 1 The subgroup of five participants with ALT/AST elevations >3 × ULN also demonstrated low fibrosis indices, consistent with the absence of clinically significant or advanced liver fibrosis even among those with transient enzyme elevations. Table S2. Pulmonary and nutritional response to ETI in patients previously treated with lumacaftor/ivacaftor (LI) vs. modulator‐naïve individuals. Although the LI group started from a lower baseline lung function, both subgroups experienced comparable improvements in ppFEV₁ and BMI, with no significant differences in exacerbation reduction. [file BCP-92-1822-s001.docx]

| **Non-invasive Fibrosis Indices (APRI and FIB-4)** | | | | |
| --- | --- | --- | --- | --- |
| **Timepoint** | **AST (U/L)** | **ALT (U/L)** | **APRI (mean)** | **FIB-4 (mean)** |
| ***Overall cohort (n = 112)*** | | | | |
| T0 | 19.7 | 20.1 | 0.197 | 0.545 |
| T6 | 25.3 | 30.5 | 0.253 | 0.568 |
| T12 | 29.7 | 33.0 | 0.297 | 0.641 |
| T24 | 21.5 | 26.7 | 0.215 | 0.516 |
| T30 | 21.4 | 24.6 | 0.214 | 0.535 |
| ***Subgroup with ALT/AST >3×ULN (n = 5)*** | | | | |
| \| *Peak* \| 126 \| 112.0 \| 1.18 \| 1.37 \| \| --- \| --- \| --- \| --- \| --- \| | | | | |

***Supplementary Table 1. Non-invasive Fibrosis Indices (APRI and FIB-4) in the overall cohort and in the subgroup with ALT/AST elevations. APRI and FIB-4 values were calculated from cohort-level mean AST and ALT at each timepoint (baseline, 6, 12, 24, and 30 months) using standard formulas. The upper limit of normal (ULN) for AST was set at 40 U/L, and the mean cohort age was 31 years. APRI was calculated as (AST/ULN_AST × 100) / Platelets (10⁹/L), and FIB-4 as (Age × AST) / (Platelets × √ALT). All APRI values in the overall cohort remained below the threshold of 0.5 used to rule out advanced fibrosis [23]. Similarly, all FIB-4 scores were far below the standard threshold of 1.45 used in clinical practice to exclude significant liver fibrosis [23]. The subgroup of five participants with ALT/AST elevations >3×ULN also demonstrated low fibrosis indices, consistent with the absence of clinically significant or advanced liver fibrosis even among those with transient enzyme elevations.***

| **ETI outcomes in Naïve vs Lumacaftor/Ivacaftor-experienced patients** | | | |
| --- | --- | --- | --- |
|  | LI Group (n=29) | Naïve Group (n=83) | p-value |
| Δ ppFEV₁ (%) | +14.8% | +15.3% | **<0.01** |
| Δ BMI (1 year) | +0.8 kg/m² | +1.5 kg/m² | **0.04** |
| Δ Exacerbations | -70% | -70% | - |

***Supplementary Table 2.*** *Pulmonary and nutritional response to ETI in patients previously treated with lumacaftor/ivacaftor (LI) versus modulator-naïve individuals. Although the LI group started from a lower baseline lung function, both subgroups experienced comparable improvements in ppFEV₁ and BMI, with no significant differences in exacerbation reduction.*
